# Innate immune response in experimentally induced bovine intramammary infection with *Staphylococcus simulans *and *S. epidermidis*

**DOI:** 10.1186/1297-9716-42-49

**Published:** 2011-03-17

**Authors:** Heli Simojoki, Tiina Salomäki, Suvi Taponen, Antti Iivanainen, Satu Pyörälä

**Affiliations:** 1 University of Helsinki, Faculty of Veterinary Medicine, Department of Production Animal Medicine, Paroninkuja 20, FI-04920 Saarentaus, Finland; 2 University of Helsinki, Faculty of Veterinary Medicine, Department of Veterinary Biosciences, P.O. Box 66, FI-00014 University of Helsinki, Finland

## Abstract

Coagulase-negative staphylococci (CNS) are in several countries the most common bacteria isolated in subclinical mastitis. To investigate the innate immune response of cows to infections with two common mastitis-causing CNS species, *Staphylococcus epidermidis *and *Staphylococcus simulans*, experimental intramammary infection was induced in eight cows using a crossover design. The milk somatic cell count (SCC), N-acetyl-β-D-glucosaminidase (NAGase) activity, milk amyloid A (MAA), serum amyloid A (SAA) and proinflammatory cytokines interleukin (IL)-1β, IL-8, and tumor necrosis factor α (TNF-α) were determined at several time points before and after challenge. All cows became infected and showed mild to moderate clinical signs of mastitis. The spontaneous elimination rate of the 16 infections was 31.3%, with no difference between species. Infections triggered a local cytokine response in the experimental udder quarters, but cytokines were not detected in the uninfected control quarters or in systemic circulation. The innate local immune response for *S. simulans *was slightly stronger, with significantly higher concentrations of IL-1β and IL-8. The IL-8 response could be divided into early, delayed, or combined types of response. The CNS species or persistency of infection was not associated with the type of IL-8 response. No significant differences were seen between spontaneously eliminated or persistent infections.

## Introduction

Coagulase-negative staphylococci (CNS) have become more important as bovine mastitis causing agents during recent years. CNS are the most frequently isolated micro-organisms in bovine intramammary infections (IMI) in many countries [1,2]. The proportion of CNS is especially high in subclinical mastitis [3,4]. The most common CNS species isolated in mastitis are *S. chromogenes, S. epidermidis, S. haemolyticus, S. simulans *and *S. xylosus *[5-7]. Typically, CNS cause mild clinical or subclinical mastitis [8].

CNS species may differ in their pathogenicity, but very little is known about their virulence factors or the immune response in IMI. One problem is that earlier studies have used phenotypic identification of CNS species, which has proven to be unreliable [9]. The duration of CNS IMI varies: infection may be spontaneously eliminated or persist over the entire lactation [10]. Infections caused by *S. chromogenes, S. simulans*, and *S. epidermidis *have been reported to persist in the quarter for longer times compared with other CNS species [7,11]. Virulence studies on CNS have mostly concerned isolates from humans. The studies have mainly focused on *S. epidermidis*, which is an important cause of nosocomial infections [12]. The best known virulence characteristic of CNS is the ability to form biofilms [13]. Biofilm-producing isolates have been reported in many CNS species [14,15]. Studies based on genotypic methods concerning CNS virulence genes are scant. Adhesion genes have been detected from CNS species isolated in bovine mastitis and in other infections [13,16]. In a recent study by Park et al. [17], superantigen genes were found in 31.2% of CNS isolates from bovine mastitis. However, genotypic methods for virulence genes of CNS are still under development and discussion [18,19].

Little information is available on the host response in CNS IMI. The only indicator of inflammation that has been investigated in relation to CNS IMI is the milk somatic cell count (SCC) [7,20,21]. In some studies, differences in the milk SCC have been observed between CNS species [7,21]. We have previously carried out a pilot experiment using a bovine mastitis model induced with *S. chromogenes*. The inflammatory reaction of the cows was mild and only 3 cows out of 5 developed a systemic response, with increased concentrations of an acute phase protein, serum amyloid A (SAA), in the blood [22]. Winter and Colditz [23] reported an increase in the concentrations of cytokines interleukin-1β (IL-1β), IL-6, and IL-8 in the milk in experimental ovine IMI with *S. epidermidis*.

The aim of this study was to investigate the bovine innate immune response in experimental *S. epidermidis *and *S. simulans *IMI. In addition, we compared the response of cows that developed a persistent infection with those that spontaneously eliminated the infection.

## Material and methods

### Study animals and study design

Each animal was infected once with *S. simulans *and once with *S. epidermidis *in a crossover study design, with a two-week experimental period and two-week wash-out period. Eight primiparous, mid-lactating dairy cows were used as experimental animals (seven of the Ayrshire-breed and one of the Holstein-Friesian breed). The cows were kept in a tie-stall barn and fed with good-quality silage and concentrate according to the Finnish feeding recommendations for dairy cows. At the beginning of the study, all the cows were clinically healthy. All udder quarters of the cows had a low milk SCC (< 100 000 cells/mL) and they were free from bacterial growth in two subsequent samplings one week before the experimental infection. The cows were randomly allocated to two groups. In the first challenge, one udder quarter of each cow was experimentally inoculated with either *S. simulans *or *S. epidermidis*. Before the infection, the mean quarter milk production in the experimental quarters was 3.96 kg/day in the *S. simulans *group and 3.56 kg/day in the *S. epidermidis *group. In the second challenge, another quarter of each cow was inoculated with the other CNS species. The infection was interpreted as a persisted infection if the inoculated strain was detected in a milk sample at the final sampling time two weeks PC, and the elimination of bacteria was confirmed when there was no growth of the inoculated strain in two successive samplings. The isolates detected in the quarters with persisting infection were confirmed to be identical with the challenge strain by ribotyping using *Hind*III restriction enzyme and oligonucleatide probes targeting the 16 and 23S rRNA encoding genes [24]. If the infection persisted over the first experimental period, the infected quarter was treated with an intramammary antibiotic at the beginning of the wash-out period (*S. simulans *with Carepen^® ^600 mg procaine penicillin G (Vetcare Oy, Mäntsälä, Finland) and *S. epidermidis *with Wedeclox mastitis^® ^1000 mg cloxacillin (WDT, Garbsen, Germany), both once a day for three days), and clearance of the infection was confirmed with bacteriological examination of a milk sample on three consecutive days on days 11, 12 and 13 after treatment.

During both study periods, one udder quarter of each cow was used as an experimental quarter and another as a control quarter. The quarters were infused through the teat canal within 30 min after the morning milking using a blunt cannula. Prior to infusion, the teat end was disinfected with chlorhexidine (5 mg/mL). After the infusion, the teat was gently closed with the fingers and the inoculation dose massaged upwards. The control quarters were infused with 7 mL of phosphate buffered saline (PBS).

The cows did not receive any medical treatment during the study periods. The Ethics Committee of Helsinki University approved the study protocol.

### Bacterial strains and preparation of the inoculate

The *S. simulans *(PM198) and *S. epidermidis *(PM221) strains had been isolated in persistent subclinical IMI from two dairy cows. The strains were primarily identified by conventional methods [25] and then with the API Staph ID 32 test (bioMérieux, Marcy l'Étoile, France) and using amplified fragment length polymorphism (AFLP). Both strains used in this study had been persisting in the cow over the entire lactation, causing a continuously elevated milk SCC in the infected quarter (for *S. simulans *max SCC 2 269 000 cells/mL, min SCC 356 000 cells/mL; for *S. epidermidis *max SCC 1 177 000 cells/mL, min SCC 154 000 cells/mL) [10]. In an in vitro study by Hyvönen et al. [26], these CNS strains showed an ability to adhere to and invade mammary epithelial cells. The *S. epidermidis *strain produced biofilm in the tissue cell plate assay (TCP) and was resistant to penicillin. The strains were stored at -80°C (Microbank^®^, Pro-Lab Diagnostics, Neston, Cheshire, UK) and were refreshed by culturing on blood agar (Tammer-Tutkan maljat^®^, Tampere, Finland) and incubating the cultures at 37°C for 18 h. Two colonies were transferred to Müller-Hinton broth and cultured at 37°C for 18 h. The density of the bacterial suspension was determined with a nephelometer at 550 nm. McFarland standards were separately calculated for both species (bioMérieux). The bacterial culture was pelleted by centrifugation and washed three times with PBS.

The suspension was diluted in saline to 0.80 × 10^6 ^cfu (colony forming units)/mL. The inoculate contained 5.7 × 10^6 ^cfu in 7 mL of saline. The suspension was cultured on blood agar plate in a dilution series and colonies were counted to determine the final inoculum dose. The infection dose used was based on experience from a previous challenge study with *S. chromogenes *[22].

### Milk and blood samples

Milk samples were taken from the experimental and control quarters for bacteriological culturing, SCC, the determination of N-acetyl-β-D-glucosaminidase (NAGase) activity, milk amyloid A (MAA), cytokines IL-1β, IL-8, and tumor necrosis factor α (TNF-α). Aseptic milk samples were collected at 24 and 0 h before the challenge and then at 4, 6, 12, 21, 27, 30, 36, 45, 54, 69, 78, 93, 102, 117, 126, and 141 h, and on the 7^th^, 10^th^, and 14^th ^day post-challenge (PC). A volume of 100 μL milk was cultured on blood agar (Tammer-Tutkan maljat) and several dilutions of the milk samples were cultured for bacterial counting on blood agar plates; the detection limit for bacterial growth was 10 cfu/mL. Colonies were identified as CNS by standard procedures [25] and occasionally with the API Staph ID 32 test (bioMérieux).

SCC was determined by a fluoro-optical method using the Fossomatic instrument at Valio Ltd Laboratories, Finland. The upper quantification limit of SCC analysis was approximately 10 × 10^6 ^cells/mL. Milk samples were stored frozen at -80 ˚C for later determinations of milk NAGase activity, MAA, and cytokines. Milk NAGase activity was measured by a fluorogenic method [27] using an in-house microplate modification developed by Mattila & Sandholm [28]. The calibrated milk sample was replaced with a control milk sample with a known 4-methyl-umbelliferon (4-MU) concentration, and NAGase activity was expressed as picomoles of 4-MU/min/μL of milk at 25 ˚C. The upper detection limit for NAGase activity was 24.5 pmol 4-MU/min/μL. Inter-assay and intra-assay coefficients of variation (CV) for the NAGase activity were 5% and 4%, respectively.

The cows were cannulated in the left or right jugular vein one day before inoculation. Blood samples were taken into vacuum glass plain tubes for five days until the 93 h PC sampling time. After that, jugular vein samples were taken by needle. Blood samples were collected at 24 h and 0 h before the challenge and then at 4, 6, 12, 21, 27, 30, 36, 45, 69, 93, 117 and 141 h, and on the 7^th^, 10^th ^and 14^th ^day PC. Serum was separated and serum samples stored at -80 ˚C for later determination of SAA and cytokine levels. The concentration of SAA was determined from serum and milk (milk SAA = MAA) using a commercial kit (Tridelta Development, Wicklow, Ireland). The concentration of MAA was determined from the milk samples taken at normal milking times. The detection limit of the kit was 0.005 mg/L. Serum and milk samples were initially diluted to 1:500 and 1:50, respectively. Dilutions of 1:1000 and 1:100 were used if the concentrations were over the range of the standard curve (75 mg/L and 7.5 mg/L). The inter-assay and intra-assay CV for the SAA analyses was < 10% and < 5%, respectively.

**Figure 1:**
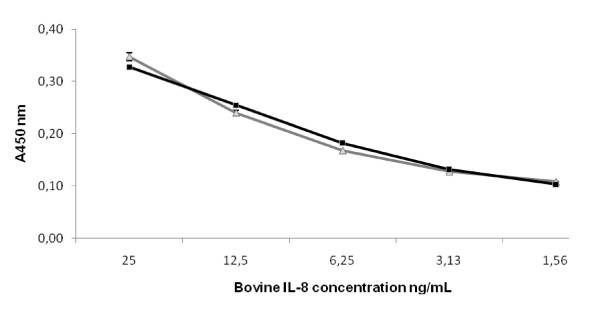
**Detection of bovine and human recombinant IL-8 using the human-specific IL-8 ELISA kit**. For each data point, the concentration of bovine IL-8 (-■-) was 100 times that of the human IL-8 (**-▲-**). The concentration of the bovine IL-8 is indicated on the x-axis. The error bars indicate S.D. (*n *= 3). A_450 _without added cytokine was 0.08 ±S.D. 0.0016 (*n *= 6).

Concentrations of IL-1β, IL-8, and TNF-α in serum and milk were determined by sandwich ELISA as described by Bannerman et al. [29,30], with some modifications. The IL-8 data was obtained using the kit's own human IL-8 standard. The data was further transformed by multiplying it with 100, since the ELISA kit detected the human recombinant IL-8 about 100 times as efficiently as bovine recombinant IL-8 (Figure 1). The specific kit, anti-cytokine antibodies and protein standards used are given in Table 1. Whey was prepared from thawed milk samples by centrifugation (16 100 × *g*, 30 min, +4°C). Capturing antibodies were adsorbed onto flat-bottom microtiter plates (655061 Greiner Bio-One Wemmel, Belgium) overnight at +4°C. The plates were washed three times with PBS pH 7.2-7.4 containing 0.05% Tween20 and blocked with 1% bovine serum albumin (BSA) (A2153 Sigma-Aldrich, St. Louis, MO, USA) for 1 h at room temperature (RT). After washing, 100 μL of non-diluted whey samples or serially diluted protein standards (Table 1) were added to the wells and analyzed in triplicate. After 2 h at RT, the plates were washed and incubated for 2 h at RT with biotin-conjugated detection antibodies (Table 1). After washing, streptavidin-conjugated horseradish peroxidase (DY998 R&D Systems, Minneapolis, USA) diluted at 1:200 in 1% BSA-PBS was added to each well and incubated in the dark for 20 min at RT. After washing, 100 μL of substrate solution containing 0.1% 3,3',5,5'-tetramethylbenzidine (T2885 Sigma-Aldrich), 0.11 M sodium acetate (443894K BDH Prolabo, Poole, UK), and 0.006% hydrogen peroxide (31642 Riedel de Haen, Seelze, Germany) was added. The reaction was stopped by adding 50 μL 2 M H_2_SO_4_. The absorbance was read at 450 nm on a microplate reader Multiscan EX (Thermo Fisher Scientific, Waltham, MA, USA). The background correction was carried out by subtracting the absorbance of PBS-coated wells from the measurements. The concentrations of measured cytokines were extrapolated from standard curves on each plate by Ascent Software 2.6 (Theorem Electron Oy, Vantaa, Finland). The intra-assay CV was < 15%.

**Table 1:** The ELISA kit, anti-cytokine antibodies (ab) and cytokine protein standards used in this study.

Cytokine	ELISA kit/antibody/protein standard	Source	Final concentration and buffer
IL-8	Human IL-8 ELISA kit	DY208 R&D Systems, Minneapolis, MN	
IL-8	Recombinant bovine IL-8	RPOIL8I Thermo Scientific, Waltham, MA	
IL-1β	Mouse anti-sheep IL-1β (capturing ab)	MCA1658 AbD Serotech, Oxford, UK	4 μg/mL in PBS
IL-1β	Recombinant bovine IL-1β	PBP008 AbD Serotech	
IL-1β	Biotin-conjugated mouse anti-bovine IL-1β (detection ab)	AHP851B AbD Serotech	2 μg/mL in 1% BSA-PBS
TNF-α	Mouse anti-bovine TNF-α (capturing ab)	MCA2334 AbD Serotech	1 μg/mL in PBS
TNF-α	Recombinant bovine TNF-α	PBP005 AbD Serotech	
TNF-α	Biotin conjugated mouse anti-bovine TNF-α (detection ab)	MCA2335B AbD Serotech	2.5 μg/mL in 1% BSA-PBS

### Clinical observations

The cows were clinically examined at every sampling. The clinical status consisted of the general attitude of the cow, appetite, body temperature, rumen function, consistency of the udder, and milk appearance. Signs were divided into three groups: systemic signs, local signs, and milk appearance. The scoring system was adapted from Anderson et al. [31] with slight modifications (scoring from 1 to 3, half numbers used as well). Signs were scored according to their severity (1 = no signs or changes and 3 = severe signs or changes). The total and quarter milk yield of the cows was measured at each milking from -2 days to 7 days PC. Measurement of the hock-to-hock distance was used to reflect possible pain during the infection at every sampling time. This method was developed by Kemp et al. [32].

### Statistical methods

After validation checks for data inconsistencies, statistical analyses were carried out using SAS version 8.2 (SAS Institute, Cary, N.C., USA). Data (IL-1β, IL-8, TNF-α, SCC, milk NAGase activity, bacterial count, SAA, MAA) were not normally distributed and logarithmic transformation was used. Analyses between CNS species and between persistent/transient infections were performed using an analysis of variance for the log-transformed area under the curve (AUC) values. When estimating the effect of CNS species, the effect of the period (first or second inoculation) and the sequence of the inoculation by each bacterial species (*S. simulans *- *S. epidermidis *versus *S. epidermidis *- *S. simulans*) were included as fixed effects in the models. In the analyses between persistent and transient infections the inoculation sequence of the bacterial species was also included in the model.

ANCOVA for repeated measurements was used for analysis of milk production and comparison of concentrations of cytokines (IL-1β, IL-8, and TNF-α) in milk between the experimental and control quarters. Possible interactions with time points were explored.

Descriptive data are presented as the mean values and SEM of groups. For all tests, *p *< 0.05 was considered significant.

## Results

### Intramammary infection

**Figure 2:**
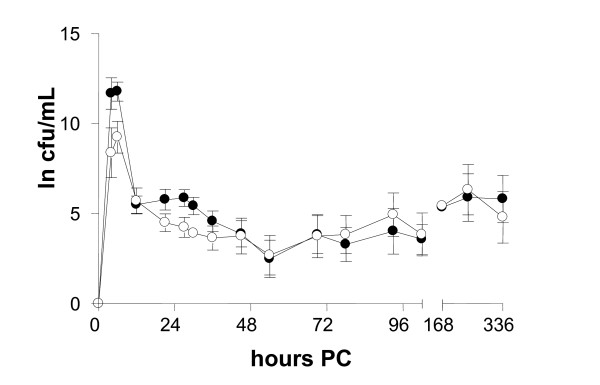
**Bacterial elimination from infected quarters in experimental *S. epidermidis *(-○-) and *S. simulans *(-●-) intramammary infection during the two-week study period (*n *= 8 + 8)**. Infection persisted in 11 out of 16 quarters. The amount of bacteria is expressed as a natural logarithm (ln) of cfu/mL.

All cows became infected with the bacterial species used in the inoculation. The bacterial count in milk samples was highest at the second sampling 6 h PC: 9.3 log cfu/mL in the *S. epidermidis *group and 11.8 log cfu/mL in the *S. simulans *group (Figure 2). After 12 h PC, the bacterial count in milk samples decreased to a constant level (3.5-5 log cfu/mL). In 11 quarters of 16, IMI remained persistent, six with *S. simulans *and five with *S. epidermidis*. In one cow, both infections were eliminated.

### Clinical signs and milk production

Systemic signs after both challenges were mild to moderate. Nine cows of 16 had fever (*t *> 39.5°C) at one time point (6 or 12 h PC). The body temperature of the cows peaked at 6 h PC; the highest mean body temperature recorded was 39.9°C in the *S. simulans *group and 39.0°C in the *S. epidermidis *group. Three cows in the *S. epidermidis *group and five cows in the *S. simulans *group were slightly depressed. Local signs in the udder were moderate; the highest score recorded was 2.5 in both groups at 12 h PC and the mean score remained under 2. Changes in the appearance of milk were mild to moderate: clots and thicker milk with a yellowish color. After 36 h PC, swelling of the infected quarters decreased; 6 quarters of 16 became even smaller compared with the other quarters of the cow.

No significant differences were found in the total milk production between infections with *S. epidermidis *or *S. simulans*, nor between infections that remained persistent or were spontaneously eliminated. The average decrease in the quarter milk production in the experimental quarters was 0.33 kg/day in the *S. simulans *group and 0.41 kg/day in the *S. epidermidis *group, when production two days before the challenge was compared with that on day 7 PC. In control quarters, no change was detected. In the acute stage 1 day PC, the drop was 1.69 kg/day in the *S. simulans *group and 1.08 kg/day in the *S. epidermidis *group; in control quarters the respective figures were 0.58 kg/day and 0.1 kg/day. The hock-to-hock distance did not significantly change during the study period.

### Systemic innate immune response: production of cytokines and SAA

**Figure 3:**
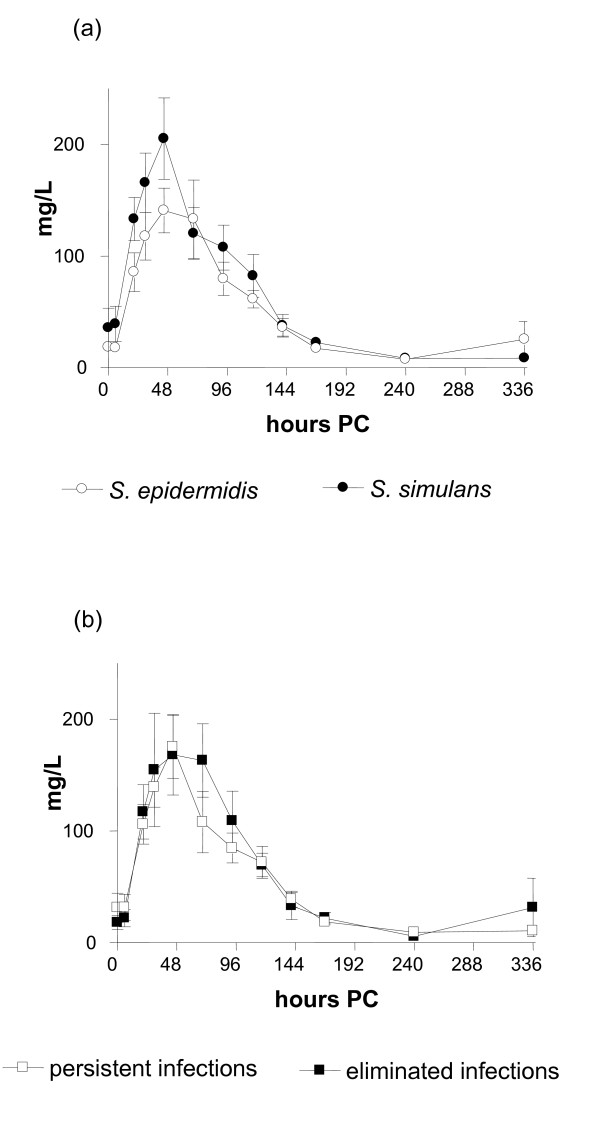
**Concentration of serum amyloid A (mean ± SEM) in bovine experimental *S. epidermidis *and *S. simulans *intramammary infection during the two-week study period shown according to the bacterial species (*n *= 8 + 8) (a) or infection status (persistent or spontaneously eliminated) (b)**. Infection persisted in 11 out of 16 quarters.

No significant changes in the concentrations of TNF-α, IL-1β, or IL-8 in serum were seen at any time point PC. No differences were seen in the concentrations of SAA between animals infected with S*. epidermidis *or *S. simulans *(Figure 3a). In both groups, the concentration of SAA peaked at 45 h PC, the maximum concentration being 205.3 mg/L (SE 36.4) in the *S. simulans *group and 140.9 mg/L (SE 19.0) in the *S. epidermidis *group. No significant differences were detected between persistent or spontaneously eliminated infections (Figure 3b). In two cows with persistent infection, SAA did not react at all but remained at the pre-challenge level. The concentration of SAA returned to below 50 mg/L within 7 days PC in all cows.

### Indicators of inflammation in the milk

**Figure 4:**
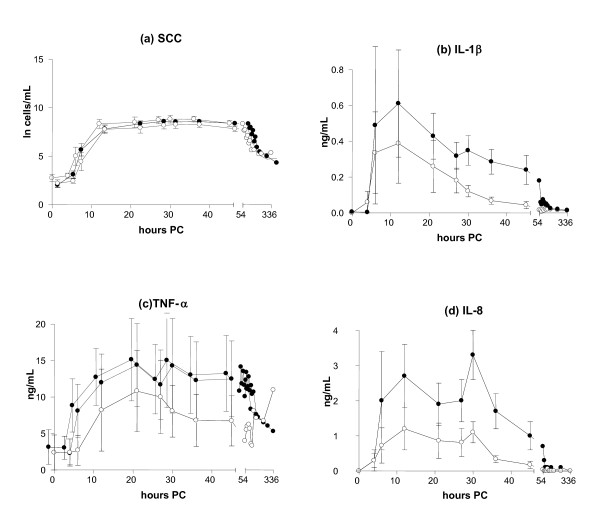
**Somatic cell count (a) and concentrations of IL-1β (b), TNF-α (c) and IL-8 (d) in the milk in bovine experimental *S. epidermidis *(-○-) and *S. simulans *(-●-) intramammary infection during the two-week study period (mean ± SEM, *n *= 8 + 8; TNF-α *n *= 8 + 7)**. Milk IL-8 (*p *= 0.04) and IL-1β (*p *= 0.03) concentrations were higher in response to infection with *S. simulans *than with *S. epidermidis*. The SCC is expressed as a natural logarithm of cells/mL.

**Figure 5:**
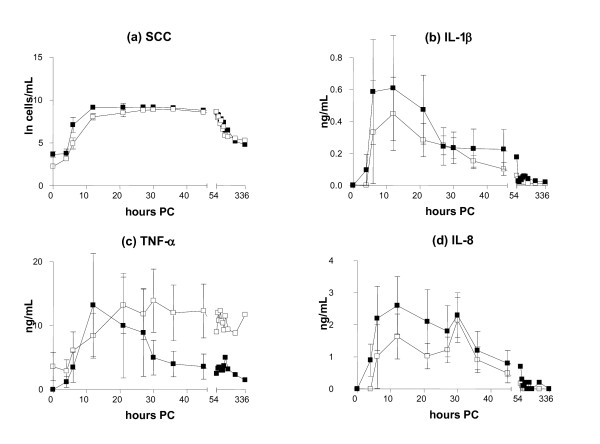
**Somatic cell count (a) and concentrations of IL-1β (b), TNF-α (c) and IL-8 (d) in the milk in persistent (-□-) and spontaneously eliminated (-■-) *S. epidermidis *and *S. simulans *intramammary infections during the two-week study period (mean ± SEM, *n *= 11 persisted infections and 5 spontaneously eliminated infections; TNF-α *n *= 10 + 5)**. The SCC is expressed as a natural logarithm (ln) of cells/mL.

The milk SCC increased until 27 and 30 h PC, and higher counts were found in IMI induced by *S. simulans *(9.3 × 10^6 ^cells/mL, SE 0.35) than by *S. epidermidis *(8.8 × 10^6 ^cells/mL, SE 1.2) (Figure 4a), with *S. simulans *tending to cause a stronger SCC response (p = 0.07). No differences were detected in the SCC between persistent and spontaneously eliminated infections during the study period (*p *= 0.52) (Figure 5a).

Increased concentrations of cytokine IL-1β were recorded in the milk of the experimental quarters as compared with the control quarters after the challenge by both bacterial species (*p *< 0.001). Milk IL-1β concentrations started to increase at 6 h PC, and the highest concentration (0.61 ng/mL, SE 0.30) was observed in the *S. simulans *group rather than in the *S. epidermidis *group (0.39 ng/mL, SE 0.22) at 12 PC (Figure 4b). In the AUC-analysis the response of IL-1β was stronger for *S. simulans *(*p *= 0.03). The peak concentration of IL-1β in the milk at 12 h PC was lower in quarters where infection persisted (0.45 ng/mL, SE 0.23) than in quarters with spontaneously eliminated infections (0.61 ng/mL, SE 0.33), but the difference was not statistically significant (*p *= 0.44) (Figure 5b).

Both staphylococcal species induced the production of TNF-α in the infected quarters compared with control quarters (*p *< 0.001), but no significant difference was seen between the CNS species (*p *= 0.25). The concentration of TNF-α in the milk peaked at 21 h PC, the maximum concentration being 14.35 ng/mL (SE 5.65) in the *S. simulans *group and 10.8 ng/mL (SE 5.6) in the *S. epidermidis *group (Figure 4c). TNF-α concentrations in the milk varied between individual cows; in four cows, hardly any response to inoculation with *S. epidermidis *was seen. One cow had a higher baseline level than the others, and one cow had an exceptionally strong reaction to *S. simulans *infection (peak level 200 ng/mL at 21-30 h PC); this cow was left out from the descriptive TNF-α data (Figures 4c and 5c). The results from this cow did not affect the results of the statistical analysis of the TNF-α response. In the quarters with persistent infection, TNF-α concentrations peaked later and remained elevated for longer than in the quarters with a transient infection, the mean maximal concentrations being 13.9 ng/mL (SE 4.9) at 30 h PC and 13.21 ng/mL (SE 8.1) at 12 h PC, respectively (Figure 5c). AUC analysis did not reveal statistically significant differences in milk TNF-α concentrations between persistent and spontaneously eliminated infections.

**Figure 6:**
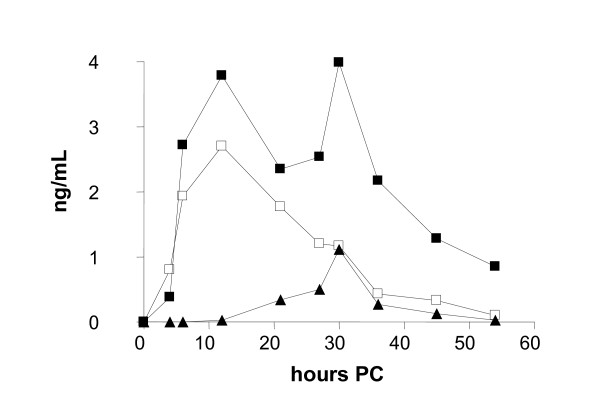
**Three different types of IL-8 response in the milk in experimental *S. epidermidis *and *S. simulans *intramammary infection during the two-week study period (*n *= 8 + 8)**. The concentration of IL-8 peaked at 12 h PC (3 challenges, -□-) or 30 h PC (7 challenges, **-▲-**) or at both time points (6 challenges, **-■-**).

The production of cytokine IL-8 was observed in the infected quarters (*p *< 0.001). The IL-8 concentration peaked at 12 h PC (3 challenges) or 30 h PC (7 challenges), or at both time points (6 challenges) (Figure 6, Table 2). Infections with a peak value at 30 h PC had milder local signs in the udder and milk, and cytokine levels were lower. The concentrations were higher in the *S. simulans *group as compared with the *S. epidermidis *group: 2.7 ng/mL (SE 0.009) versus 1.2 ng/mL (SE 0.006). In the AUC-analysis IL-8 response was stronger for *S. simulans *(*p *= 0.04) (Figure 4d). The average peak IL-8 concentration in the milk of the quarters with a persistent infection was lower (2.1 ng/mL, SE 0.007) than in the quarters that cleared the infection (2.6 ng/mL, SE 0.009), but no significant difference was observed in the AUC analysis (*p *= 0.27) (Figure 5d).

**Table 2:** Distribution of different IL-8 response types in bovine *S. epidermidis *and *S. simulans *experimental intramammary infection (*n *= 8 + 8).

	*S. simulans*		*S. epidermidis*		
IL-8 type	Spontaneously eliminated infections	Persistent infections	Spontaneously eliminated infections	Persistent infections	Total
type 1		1	2		3
type 2		2	1	4	7
type 3	2	3		1	6

The differences of IL-8 types are presented in Figure 6.

MAA started to increase at 21 h PC and concentrations peaked at 45 h PC (94.0 mg/L, SE 45.5) in *S. simulans *infection and at 30 h PC in *S. epidermidis *infection (62.3 mg/L, SE 35.6), with no differences between the CNS species (*p *= 0.15) or between persistent and spontaneously eliminated infections (*p *= 0.99).

**Figure 7:**
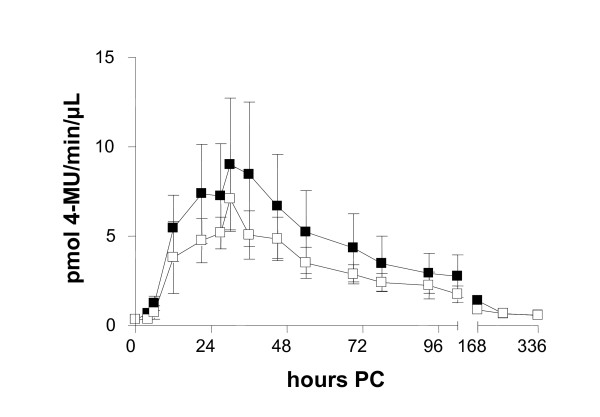
**Milk NAGase activity in experimental *S. epidermidis *and *S. simulans *intramammary infection (*n *= 8 + 8) during the two-week study period (mean ± SEM). Infection persisted in 11 out of 16 quarters**. No significant differences were seen between persistent (-□-) and spontaneously eliminated infections (-■-).

Infections by the two species resulted in similar levels of milk NAGase activity; peak activities were seen at 30 h PC 11.03 pmol 4-MU/min/μL (SE 2.72) in the *S. simulans *group and 4.35 pmol 4-MU/min/μL (SE 0.76) in the *S. epidermidis *group. No differences were seen between persistent and spontaneously eliminated infections (*p *= 0.54) (Figure 7).

## Discussion

Experimental infection with two CNS, *S epidermidis *and *S. simulans*, caused clinical mastitis with a short period of mild to moderate signs. The local immune response manifested as increased concentrations of cytokines and indicators of inflammation in the milk, which were more intense in IMI induced by *S. simulans *than by *S. epidermidis*. Systemic signs were mild or moderate and did not differ between the infections. *S. simulans *infection resulted in a slightly stronger innate immune response. This could support the assumption of *S. simulans *as a more specific mammary pathogen than other CNS species. In a study on the ecology of CNS, *S. simulans *was mainly detected in the milk of quarters with IMI, but only seldom in extramammary sites such as teat and udder skin, contrary to other CNS species [33]. *S. epidermidis *is less common in bovine IMI and is suspected to mainly originate from human sources [34]. Even though the challenge strains were isolated in subclinical mastitis, they caused short term clinical signs. This may be related to the experimental model with a relatively high inoculum dose; in field conditions CNS infections probably progress more slowly and mostly without clinical signs. However, spontaneous CNS infections can also cause clinical mastitis [5].

Pathogens invading the mammary gland or their metabolites activate the production of pro-inflammatory cytokines such as IL-1β, TNF-α, and IL-8. This is the first study to determine the cytokine response in bovine IMI caused by CNS. Infection triggered an IL-1β, IL-8, and TNF-α response in the mammary gland, while the systemic levels of these cytokines remained unchanged. A systemic acute phase response was, however, induced, because increased concentrations of SAA were recorded. Individual cows had a rather similar SAA response in both challenges (data not shown). Two cows with persistent infections had no systemic acute phase reaction, as their SAA concentrations in the blood did not increase.

Concentrations of IL-1β in the milk were comparable with those seen in experimental IMI induced by *S. aureus *and *E. coli *[29,35]. However, the peak levels were reached earlier, possibly due to the large inoculation dose used in this experiment.

IL-8 is a chemotactic cytokine, which has a long lasting effect on neutrophils [36]. In this study, the IL-8 responses of the cows differed between challenges. The responses could be divided into early or delayed responses, or combined responses including two peaks. No association was seen between the IL-8 response and the CNS species, persistency of IMI or individual cow response. The first peak in the IL-8 concentration was recorded at the same time, around 12 h PC, as seen in earlier studies on *E. coli *and other Gram-negative bacteria [29,36]. In experimental IMI induced by the Gram-positive bacterium *Streptococcus uberis*, a delayed IL-8 response at 30 h PC was reported [37]. On the contrary, no IL-8 response was seen at all in two studies on experimentally induced *S. aureus *mastitis [29,35], but in a third study, IL-8 transcripts were detected in the milk [38]. In those studies, the inoculation doses of *S. aureus *were much lower than in our study, from 50 to 100 CFUs. In ovine experimental mastitis induced by *S. epidermidis*, early and late peaks of IL-8 were detected in the milk, in accordance with our study [39]. However, the results from different experiments are difficult to compare because of differences in inoculation doses, bacterial species and analytical methods, and the large variation in individual responses of the cows also affects the results. In this study, milk samples were frozen before centrifugation. Thus, both extracellular cytokines and those released from ruptured cells contributed to the observed concentrations. If the milk sample is centrifuged right after sampling, whey consist only extracellular cytokines.

Biofilm production is a well known virulence factor [13]. *S. epidermidis *is a typical biofilm producer, as also was our experimental strain [40]. Another characteristic associated with the virulence of a pathogen is its capacity to enter the host cells. In in vitro studies using bovine mammary epithelial (BME) cells, internalization of CNS species has been demonstrated [41]. In a study by Hyvönen et al. [26] on BME cells, the first stage of internalization, adhesion to the cells, was quite similar between CNS isolates and *S. aureus *isolates, but subsequent invasion was weaker. The CNS strains used here for experimental challenge were included in that study; the capacity for adhesion to BME cells of the *S. simulans *strain was significantly stronger than that of *S. epidermidis *strain, which could explain the differences seen in the innate immune response of the cows in the present study.

Infection was eliminated from 5 quarters out of 16, i.e. the spontaneous elimination rate was 31.3%. In our pilot study with *S. chromogenes*, 4 out of 5 cows eliminated the infection [22]. No differences between the two CNS species in this respect were seen here, as both *S. simulans *and *S. epidermidis *were able to cause persistent IMI. In the end of the study period, the concentration of TNF-α in the milk was higher in the quarters with persistent infections, possibly due to the bacteria present in these quarters.

In conclusion, experimental IMI with *S. simulans *and *S. epidermidis *induced cytokine and APP responses in milk. A systemic APP response was detected and cows had mild clinical mastitis. *S. simulans *caused a slightly stronger innate immune response. Spontaneously eliminated infections tended to have stronger responses, although the differences were not statistically significant. With species-level diagnostics and more knowledge on the virulence of CNS species, control measures could be targeted to the more harmful representatives of the group. Fairly ineffective results with control strategies in CNS mastitis problem herds suggest that critical points to prevent CNS intramammary infections are still unknown [21,42].

## Competing interests

The authors declare that they have no competing interests.

## Authors' contributions

HS and ST carried out the experimental challenge and bacteriological studies. HS performed milk NAGase, MAA and SAA determinations and drafted the manuscript. TS performed cytokine analyses and co-drafted the manuscript. SP and AI participated planning and coordinating the study and critically revised the manuscript. All authors participated in study design and approved the final manuscript.
